# Editorial Notes

**Published:** 1916-12

**Authors:** 


### Editorial Notes.

---

**The late Sir Victor Horsley.** A PERSONAL note from one who was closely associated with this great-hearted colleague in Mesopotamia, where his patriotic service cost him his life, cannot fail to interest all our readers. Captain Carey Coombs writes as follows :—

"The first newspaper I bought in England on landing at Southampton on July 23rd contained the news of Sir Victor Horsley's death. I had last seen him on June 7th, the day before I left Amara on the long homeward trip. He was full of plans as to what he was going to do after the war. One of his dearest projects was an inquiry into the arrangements for supply and transport in the Indian Army, particularly in its medical aspects. On his way out to Mesopotamia he had visited India, and the visit had not been forgotten in Bombay several months later. When he reached Mesopotamia there was not much surgical work doing, but he threw himself with his accustomed zeal into the task of collecting data for a report on the conditions of the medical services in that country. Those who knew him can guess at a little of the attitude of mind which he adopted in regard to the management of the transport problem on the Tigris.

"I saw a good deal of him during my last few weeks at Amara. It was interesting to see how completely he had captivated even those who expected to find themselves antagonistic to him. They had known of his views, but had no knowledge of his personal charm.

"From the accounts I have read, the heat must have 'got' him while walking over an open stretch near our hospital. He persisted in going about his business out of doors during the heat of the afternoon, a thing which no one could do with impunity. When we ventured to remonstrate with him he declared that the heat agreed with him, and that his experience of sun in Egypt and Gallipoli had inured him to it. Mayhap he believed this, and thought himself able to defy or ignore the sun's effects. We had some interesting discussion as to the actual factor in the sun's rays responsible for its deleterious effects on man. That it was not the heat of it was suggested, so he argued, by the fact that sun-burn is more likely to follow exposure to sun-glare at high altitudes than in tropical plains.

"Once I asked him, 'When are you going home?' To which he said, 'What do you mean, my dear fellow? I am here for as long as I am wanted.' I had an idea that he had come out for a definite period, to return later to England to report. Of course, he could have gone home if he had wished, but he preferred to face the discomfort and danger of the hot season, believing that therein lay his duty. The impression left upon my mind of his utter forgetfulness of himself, his absorption in the welfare of that great gentleman the British soldier, will never be effaced. Of his kindness to me personally I say little, but remember much. Not only in Amara (which he once described in a letter as a 'burning mud-flat, surrounded by cholera and dysentery') is there a momunent sacred to his memory, but also in the minds of that vast host of his fellows whom he was never weary of serving with all his great gifts."

**The L.G.B. Scheme for the Treatment of Venereal Diseases.**

Great issues hang on the methods that may now be instituted with a view to giving practical effect to the conclusions of the Royal Commission on Venereal Diseases. The action taken by the Local Government Board, and the regulations laid down in their scheme, constitute a commendable endeavour to lose no time in arriving at a satisfactory solution of a very difficult problem, and there need be no doubt that the medical profession will do their utmost to support the essential principle of the scheme, viz., an endeavour to stamp out, as far as possible, the ravages of diseases coming under the term venereal.

It would be superfluous to relate in detail the regulations referred to, they have already been before the medical profession, as well as available for the layman for some months. But there are certain questions involving both the medical practitioner and the voluntary hospital system that merit our careful and considerate attention before the scheme matures.

The outstanding features of the scheme may be summarised :—

*a.* The provision of laboratory facilities for diagnosis and for guidance in treatment, and "the laboratory should be in charge of a skilled pathologist of experience in this particular work." The arrangements for carrying out the scheme are to be made by the County Councils of every county and borough, subject to the approval of the Board, and the Councils are to consult their Medical Officers of Health as to the arrangements best suited to the circumstances of the areas concerned. But responsibility for the measures to be adopted is to be undertaken by the State.

*b.* The treatment should be available for the whole community, and the Commissioners recommend that local authorities should approach general hospitals with a view to securing facilities for treatment. "In negotiating with the authorities of hospitals the Council should make it clear that the treatment provided must be available for all comers, irrespective of the place of residence or the means of the patient." The Government grant of 75 per cent. of the cost of the approved schemes of treatment has been fixed at this high proportion in order that the facilities provided under these schemes should be available for the whole population.

*c*. In all cases it will be important to arrange that the clinics are not specially designated as for venereal diseases, and that nothing is done to distinguish the patients who attend for treatment of these diseases.

Of the scientific exhaustive and painstaking effort of the Royal Commission we have only a feeling of admiration and thankfulness. Nothing could more clearly demonstrate the need for a very special effort to cope with evils that have made disastrous inroads in the health and vitality of the community. It is, we trust, an earnest only of further *State* efforts to meet in the open and to overcome successfully the ravages of disease at any wise cost, and the recognition that "no short-sighted parsimony should be permitted to stand in the way of all means that science can suggest for guarding the present and future generations, upon whom the restoration of national prosperity must depend," is a great advance on the idea that has hitherto prevailed, viz., that it is a charity only, rather than sound political economy, to relieve sickness and suffering in the poorer members of the community.

Now we are to realise that it is cheaper that we and our offspring should be guarded against venereal infection, and worth almost any price to ensure this as far as possible, and it is high time that such excellent sense should be brought home to the community. But at what cost? Great issues are at stake, and hence some feel that the evil must be remedied at any cost.

The voluntary hospitals are the creations of the charitable public to provide what the State has failed to provide for the necessities of the sick poor, and it is essential that they should be restricted to the poor, *i.e.*, to those who are not in a financial position to provide for their own medical needs that which the State—directly or indirectly—has not afforded. If a patient is suffering, say from cancer, or heart-disease, unless it be through the poor laws and their infirmary wards, the State makes no provision whatever for his treatment. But the charitable purse has provided for all such who cannot afford to do so for themselves; though every well-regulated hospital declines to open its doors to those who can well afford to pay for adequate medical attendance, since to do otherwise would be a fraud. Why, then, should the wealthy patient who contracts venereal disease be singled out as the one class of patient for whom the fundamental basis of voluntary hospital systems is to be set at naught. If it is conceded that the medical charities supported by voluntary subscription is open to allcomers suffering from venereal diseases, by what reasoning are the doors to be closed to any well-to-do patient whose eyes, ears, lung or stomach is a source of trouble or who may be suffering from inebriety. Doubtless the argument will be put forward that treatment for venereal diseases is henceforth a State provision, and that all other complaints, unless it be a tuberculous or infectious fever, are cold-shouldered by the State or other public authority, not because the urgency is less acute for the individual, but on the ground that cancer of the stomach is a question for the individual, while gonorrhæa may spread. The real reason for grafting the State venereal disease clinics on to the voluntary hospitals is that the good name of these charities may serve as a convenient cloak for the susceptibilities of venereal patients who are not to be compelled to notify their disease.

The Royal Commission's report refers to another important question, the widespread existence of unqualified persons or quacks engaged in the treatment of venereal diseases and states that the Commission "has no hesitation in stating that the effects of unqualified practice in regard to venereal diseases are disastrous, and that, in our opinion, the continued existence of unqualified practice constitutes one of the principal hindrances to the eradication of those diseases." The medical profession has long been of that opinion, and now that the State is to make the much-needed provision for the treatment of venereal diseases without unworthy suspicion of ulterior motives, we can again urge the wisdom of forthwith making it penal for any unqualified person engaging in such disastrous practices, *i.e.*, disastrous to the individual and to the community. Yet this obviously wise course is to be left for future consideration, and we are to rest content with an inactive, pious opinion, instead of clear-cut legislation on this point.

While it is well to set forth clearly objections to the scheme on which the Government have decided to take action, there should be no half-hearted support or any wavering in endeavour to make the scheme successful by those hospitals which undertake their share in the work, and as the scheme is open to modification after it has been in operation for a limited period, practical experience may enable us to arrive at useful conclusions for our future guidance, and it is better to make mistakes than to hesitate unduly to embark on a work that is a matter of very grave urgency and of momentous national import.

By no means secondary in point of value is the necessity for maintaining the interest of general practitioners in the scheme when launched. We might have referred to the *interests* of the practitioners, for that would only be bare justice, but prefer to enlarge on this aspect of the question as it concerns the general public rather than that of medical practitioners. A moment's reflection must make it clear to every thinking man that any scheme which tends to the exclusion of the general practitioner in dealing with the treatment of venereal diseases is foredoomed to failure in the attainment of the essential aims of the Royal Commissioners' report, and that this was the view of the Commissioners no one who reads the report can doubt. Every possible facility for the general practitioner to obtain adequate training in the latest advances in diagnosis and treatment is to be made available for all who feel the need of such assistance, and their patients will correspondingly benefit.

But as teachers must be experts, the special clinics will have to be under the charge of those who are prepared to devote a large share of their time to this special work. The British Medical Association has recommended that the commencing salaries of whole-time medical officers of clinics set up under the schemes be not less than £750 per annum, exclusive of expenses and clerical assistance, and where a whole-time assistant is necessary to a part-time senior medical officer, he should be paid not less than £350 per annum (non-resident); and for part-time senior medical officers of these clinics the rate for one or two sessions per week (not exceeding 2½ hours a week each) should be £3 3s. per session; or, if there be three or more such sessions per week, £2 12s. 6d. per session should be paid—that is in London. Curiously enough, the British Medical Association deduct one guinea per session from these London rates if the clinic is a provincial one. We demur to the suggestion that the rate should be reduced for increasing numbers of sessions per week, and really three guineas for 2½ hours work by an expert is little enough, and is intended, no doubt, to indicate that the best expert advice is not to be available, or if it is, that the State payment should be inadequate remuneration. But we demur all the more strongly to the remarkable suggestion that the work, if done in London, is worth 33 per cent. more than in Bristol. We wonder what difference would be made between male and female, if say a lady doctor took up the work in so far as it concerned women and children. We trust that, once for all, the provinces will let it be known that they will not concur in these invidious distinctions in the payment of London and provincial medical work. It is archaic to suggest that the same practitioner who gets £315 a year in London is only worth £210 in Bristol, Manchester, Lverpool, or Birmingham. To admit such a principal in connection with this scheme would lead to grave misfortune.

That the scheme will go through is no longer a matter for discussion. We are indebted to Mr. Bishop Harman, F.R.C.S., Hon. Secretary of the Metropolitan Counties Branch of the B.M.A., for the following information: "There are no less than twenty-two hospitals in the London County area which are arranging to work the home counties scheme as from January 1st, 1917, viz.: St. Bartholomew's in the City of London, and for the L.C.C., Bucks., Essex, Hertford, Middlesex, Surrey, Kent, and the county boroughs of Croydon, East and West Ham, twenty-one hospitals come into the scheme, viz.: Charing Cross, Great Northern, Guys, Great Ormond Street (Children's), King's College, Lock, London, Middlesex, Miller's at Greenwich, New Women's, Royal Free, Seamen's at Greenwich, South London Women's, St George's, St. John's, St. Mary's, St. Paul's, Red Lion Square, St. Thomas', University College, West London, Westminster."

**Death of Sir George White, Bart., President of the Bristol Royal Infirmary.**

The sudden death on November 22nd of Sir George White, a powerful factor in the Bristol Medical Charities, was not only a cause of genuine sorrow to his many friends, but deprives our oldest Medical Charity of a brilliant and enthusiastic organiser, at a time when perhaps as never before such qualities as the late President possessed in singular degree are needed. Few men have presided over and guided so many large concerns as did Sir George White, fewer still possessed in equal measure the power of foreseeing and of initiating schemes to meet future events. When tramway lines were first laid down in Bristol, he as a young man realised the possibilities of their development far and wide; in later years he it was who initiated the Bristol Aeroplane Works, and despite continual rebuffs from our Government, poured enormous sums into the concern to develop works which are now a valuable asset in the present war; hence when he accepted the Presidency of the Bristol Royal Infirmary not many years since, he very soon realised that it was absolutely essential to provide the long-needed new housing and equipment which he induced the Governors to sanction. To do this it was necessary to expend nearly all the accumulated endowment of the Charity; but having thus given it a splendidly-equipped new building, the King Edward VII. block, he set to work to organise support by subscribers, till the increased subscription list far exceeded the former annual income from endowment. Thus while the dead hand built the Institution the living hand supports it.

Those who are associated with the Royal Infirmary will greatly miss his genial presence and that of Lady White—who died but a year ago—for many years to come; and we desire to convey to his son and daughter and other members of the family our sincere condolences for the loss which in a very real sense we share.

